# The Prevalence of Cancer in Dutch Female Patients with Idiopathic Scoliosis Compared with the General Population

**DOI:** 10.3390/jcm13092616

**Published:** 2024-04-29

**Authors:** Reinout R. O. Heijboer, Johan L. Heemskerk, Sigrid N. W. Vorrink, Diederik H. R. Kempen

**Affiliations:** 1 Department of Orthopedic Surgery, OLVG, 1091 AC Amsterdam, The Netherlandss.n.w.vorrink@olvg.nl (S.N.W.V.); d.h.r.kempen@olvg.nl (D.H.R.K.); 2 Department of Radiology and Nuclear Medicine, Amsterdam University Medical Center, 1012 WP Amsterdam, The Netherlands; 3 Department of Orthopedic Surgery, Amsterdam University Medical Center, 1012 WP Amsterdam, The Netherlands

**Keywords:** idiopathic scoliosis, radiological exposure, cancer prevalence, orthopedic surgery

## Abstract

**Background and Objectives:** Epidemiological studies have demonstrated the potential oncogenic effects of cumulative radiation exposure, particularly during childhood. One group experiencing repeated exposure to radiation at an early age for multiple years is patients treated for idiopathic scoliosis (IS). This study aimed to determine the relationship between childhood radiological exposure and adult cancer prevalence in children treated for IS. **Materials and Methods:** Data from 337 predominantly female patients treated at our hospital between January 1981 and January 1995 were gathered and compared to the Dutch national cancer rates. The standardized prevalence ratios for cancer in IS patients were compared with the cancer prevalence rates from the general Dutch population. **Results:** The overall cancer prevalence in women was 5.0%, with no significant difference compared to the general population (*p* = 0.425). The results of this study do not suggest that female patients treated for idiopathic scoliosis during childhood have an increased risk of cancer later in life. **Conclusion:** Despite being the largest recent study in its field, the modest participant number limits its ability to draw conclusions. However, the detailed data collected over a long observation period, alongside data from a period with comparable radiation rates, contributes to refining clinical practice and laying the groundwork for future systematic reviews.

## 1. Introduction

Radiation, such as X- and gamma rays, is known to cause cancer. Most of the evidence for this comes from research on people exposed to high doses of radiation—e.g., atomic bomb survivors in Japan [1,2]. There is less evidence on the cancer risk resulting from the lower levels of radiation used in radiographic imaging. Nevertheless, most scientists and regulatory agencies, such as the International Commission on Radiological Protection (ICRP), agree that even a small radiation dose increases cancer risk. There is no threshold below which radiation exposure is considered totally safe (the so-called linear, non-threshold hypothesis) [3]. However the ICRP states that emerging results concerning radiation-related adaptive responses, genomic instability, and bystander effects suggest that the risk of low-level exposure to ionizing radiation is uncertain, and a simple extrapolation from high-dose effects may not be completely justified in all instances [4]. Multiple studies have reported a lower prevalence of cancer in individuals who are occupationally exposed to low doses of radiation when compared to the general population. These findings suggest that low-dose exposures may have a protective effect, a phenomenon known as hormesis, which may decrease the risk of developing cancer [5]. The controversy surrounding the potential risks of low-dose radiation makes it imperative to conduct studies that can determine whether such exposure can increase the risk of developing a malignancy. Research has demonstrated that children may be more vulnerable to the harmful effects of radiation than adults due to having more dividing cells on which the radiation can act and a longer period in which to develop cancer [1,6].

A group of patients frequently exposed to radiation during childhood comprises patients with idiopathic scoliosis (IS). IS is a complex three-dimensional spine deformity with a reported prevalence of 0.5–5.2% in children and occurs most frequently in females [7,8,9]. The treatment varies according to the age of onset, the extent of skeletal development, the potential for progression of the curve, and its severity. It is difficult to predict whether a curve will progress during growth and what the rate of progression will be. For these reasons, imaging with plain spinal radiographs remains the gold standard for its diagnosis and management. This leads to repeated imaging during follow-up, resulting in a relatively high cumulative radiation dose at an early age.

Previous studies have suggested a correlation between frequent exposure to radiation and the risk of cancer in IS patients and found a higher incidence of breast and endometrial cancer compared to the general population [10,11,12]. However, this increased cancer risk may be due in part to the outdated imaging techniques used before 1965 with higher radiation doses and less concern for the effects of radiation [13]. Although important, the majority of studies conducted after 1965 tend to suffer from limited sample sizes and/or focus exclusively on particular cancer types. Further research is needed to determine whether the results of these studies can be replicated in a different cohort and to further elucidate the association between cumulative radiation dose and the prevalence of cancer in patients treated for IS. Therefore, the current article investigates whether exposure to radiation during past scoliosis treatment could be associated with an increased prevalence of cancer relative to the general population.

## 2. Materials and Methods

This cross-sectional study was part of a long-term clinical outcome assessment of idiopathic scoliosis patients from our institution (OLVG). Local ethical approval was obtained before the start of the study (WO 15.017). The inclusion criteria consisted of (1) idiopathic scoliosis patients, (2) who consulted our hospital between January 1981 and January 1995, (3) between 4 and 18 years of age at the time of first evaluation with spinal X-ray. The exclusion criteria were (1) inadequate knowledge of the Dutch language or (2) unwilling to participate in the study. Childhood medical information, such as age at first and last radiograph, age at menarche, body mass index (BMI), Cobbs angle, juvenile or adolescent IS, scoliosis treatment, and operation reports, was obtained from the original medical records. Radiology reports, radiographs, and radiology logbooks were reviewed to extract the following information: date of radiograph, projection (e.g., full spine, thoracolumbar spine, pelvis), and view (e.g., anteroposterior, posteroanterior, lateral). Eligible patients were identified in a single-center registry established in the 1970s, traced, and contacted by phone or mail to inform them about the study. Before contacting the patients, hospital records were updated using the Dutch Personal Records Database to check whether patients were still alive and avoid the risk of contacting family members of deceased patients. After obtaining informed consent, digital questionnaires were sent including questions about cancer history and information on various medical conditions and relevant risk factors (e.g., smoking status). For deceased patients, the Netherlands Comprehensive Cancer Organisation (IKNL) was contacted to obtain information on the history of cancer. Nationwide age-specific cancer rates were requested from the IKNL and compared to our cohort. The IKNL is a population-based mandatory national registry that records all malignancies based on notification by the National Pathology Archive and hospital discharge registries.

### Statistical Analysis

Nationwide age-specific cancer rates from the IKNL were compared to the prevalence of cancer in our cohort of IS patients using a standardized prevalence ratio (SPR; ratio of observed prevalence to expected prevalence). If a person developed cancer multiple times during our follow-up period, only the first event was included in the analysis, similar to the IKNL database. SPRs were computed by dividing the number of observed cancer rates by the number of expected cancer rates. An SPR of 1.00 indicates that patients with scoliosis had the same cancer rate as the general population, whereas an SPR of 1.60 indicates a 60% higher rate. Prevalence rates from the IKNL were taken from 10 years of age because the majority of our population (86%) had their first X-ray after this age. The analysis was carried out solely on the female participants of our cohort, as they constituted the majority of the sample. This decision was made to ensure a representative analysis and to avoid potential confounding factors that could arise from including the small number of male participants. A Z-test for independent proportions was used to analyze the possible differences in proportions between cancer rates. The difference in cancer rates between operated and non-operated patients was compared using an independent *t*-test. The number of X-rays between these groups was compared using a chi-square test. Analyses were performed with Stata^®^ 13.0 (StataCorp LP, College Station, TX, USA), and *p* values less than 0.05 were considered significant.

## 3. Results

The database contained 567 eligible patients based on the inclusion criteria. During the update of hospital records using the Dutch Personal Records Database, it appeared that 14 patients were deceased. Sixty-five patients were excluded because we could not find any old data on their radiology history. Of the remaining patients, 420 could be traced and were contacted to participate. Follow-up was complicated by the fact that most patients were last seen as teenagers. Consequently, 323 patients were included in this study. The cancer history of the 14 deceased patients was checked with IKNL and were included. There were 2869 radiographic examinations (61%) for which it was not possible to differentiate between the anteroposterior and posteroanterior view. A careful review of the data for the other films and protocols from those time periods suggested that the posteroanterior view was used almost exclusively throughout the study period to reduce the radiation dosage on the breasts. The mean age at diagnosis was 11.4 (SD 2.9) years, and the mean follow-up time was 31 (SD 6.5) years. Patients were on average 44 (SD 6.6) years old at final moment of follow-up (age at questionnaire, date of death, or date of cancer). Patients had a median period of 5.8 (IQR 3.1–10) years between their first and last X-ray and received a median of 14 (IQR 8–19) radiographs during childhood (Table 1). The total cancer rate in our sample of IS patients was 5.6% (19/337), including two patients who had developed a second malignancy after the first onset (Table 2). Two patients with breast cancer had a recurrence: one patient after 2.5 years and the other after 6.5 years. One patient with cervix carcinoma developed primary papillary renal cell carcinoma after 2 years, and one patient with astrocytoma developed non-small-cell lung carcinoma 15 years after the first onset. These recurrences were not included in the analysis or prevalence rate, similar to the IKNL database. In total, fourteen patients were deceased, of whom one died due to breast cancer (female), one died of anal carcinoma (male), and the rest of non-cancer disease.

**Figure 1:**
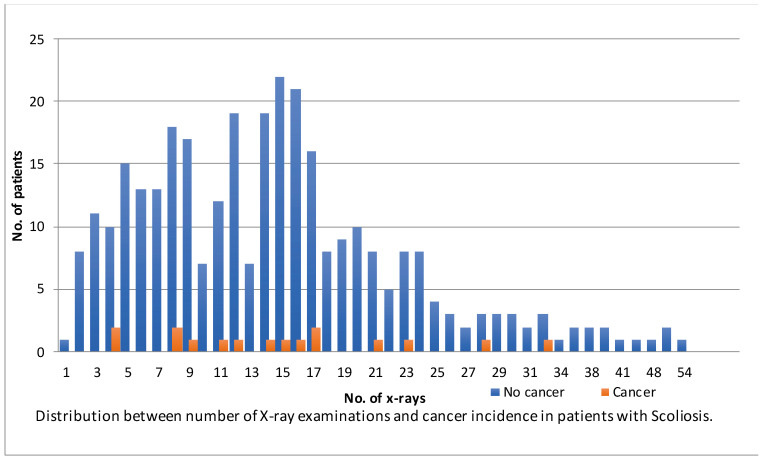
Relationship between total number of radiographs and number of patients who did and did not develop cancer.

Because IS primarily occurs in women, the number of men in this study was limited. Therefore, prevalence rates were only compared for female patients. Basal cell carcinoma of the skin was excluded from the analysis since this was not registered in the IKNL database. Our analysis of the female cohort did not reveal any statistically significant differences in the proportions of cancer rates when compared to the general population: observed 15/298 (5.0%) compared to the expected 4.8% (SPR = 1.10; *p* = 0.425 in the one-proportion Z-test). The prevalence of cancer between the Dutch female population and the women in our cohort is illustrated in Table 3. Patients who underwent surgery received more radiological examinations than patients treated non-operatively (17 IQR 12–24 vs. 13 IQR 7–17; *p* < 0.001). There was no statistically significant difference found in the prevalence of cancer between patients treated surgically and non-operatively (6.7% vs. 3.4%; *p* = 0.104). As depicted in Figure 1, no correlation was observed between the total number of radiographs and the patients who developed cancer (*p* = 0.90).

## 4. Discussion

The general consensus is that radiation causes damage and above a certain dose increases the risk of cancer. Especially in younger patients, multiple radiograph exposures have become a source of concern with several studies showing that harmful effects can be linked to ionizing radiation. However, the cancer risk from low-dose medical imaging is debated and mainly extrapolated from much larger radiation loads [3,5]. Therefore, this study investigated the prevalence of cancer in a cohort of patients who had a relatively high radiation exposure as children or adolescents. The results of this study suggest that female patients who were treated for idiopathic scoliosis during childhood did not show an increased risk of developing cancer later in life (*p* = 0.425). It is worth noticing that among surgically treated patients who underwent more radiological examinations, there was not a significantly higher prevalence of cancer (*p* = 0.104).

In contrast to our results, previous studies have reported an increased risk of cancer of IS patients [11,12,13,14,15,16,17]. In a Danish study, IS patients had a relative risk of 4.8 (CI 2.3–5.8) for developing cancer compared to the national population collected from the NORDCAN database [12]. Although our cancer rate was even higher (19/337 patients, 5.6%) than the Danish study (9/211 patients, 4.3%), the method of selecting the reference groups and thus the conclusions differed. After consultation with the IKNL, an approach with standard prevalence ratios (SPR) was used. This enabled a more accurate comparison by accounting for age differences in the population and offered a more nuanced assessment of the actual relative risks. The incidence of cancer varies significantly across different age groups. Direct comparisons do not account for these variations, potentially resulting in misleading interpretations of the risk of cancer in the IS population. Using a standardized difference helps eliminate the potential bias introduced by comparing age-specific cancer rates directly across age groups. This is especially important when dealing with diseases that have a distinct age distribution. Moreover, when the national cancer incidence at a specific age is taken as a reference group, people who died at a younger age are not taken into account anymore. For example, the chance of a Dutch 10-year-old girl developing cancer before the age of 45 (3.77%) is higher compared to the cumulative incidence of cancer for females between 40 and 44 years old (0.7%). Since the risk of cancer in the reference population is higher using this method, no significant difference was found.

A similar method to ours was used by other studies investigating the risk of breast cancer and breast cancer mortality among scoliosis patients diagnosed before 1965 [10,15]. These studies found a higher standardized mortality rate of 1.52 (95% CI 1.0–2.2) [10] and a 1.8 (90% CI 1.0–3.0) ratio between expected and identified breast cancer cases [15] in scoliosis patients compared to the normal population. Both studies found that breast cancer risk increased with the number of X-rays and with cumulative dose. However, both studies involved patients treated between 1935 and 1965, and doses of X-rays from spinal radiographs were lowered by about 90% by modernizing equipment in the 1990s [18]. In addition, the risk may be overestimated because other underlying causes of scoliosis and carcinogenic factors other than radiation were included [18,19]. Some studies investigating cancer risk relied on estimated calculations, to project the theoretical risk of cancer, without objectively having recorded the cancer incidences in their population [11,14,16,18,20]. Although these studies have created awareness and prompted various adjustments in clinical practice in the past, it is important to improve our current study methodologies and gather data from periods with radiographic equipment and protocols exhibiting comparable radiation exposures to the present. This study provides a necessary counterbalance, contributing to the broader scientific conversation and offering nuanced insights that challenge existing norms.

Most older studies discussing the harmful effects of radiation produce dramatic results describing outdated techniques [13]. The patients included in this study were already managed using many current practices like limiting the number of radiographs and a reduction in the radiation dose and optimized protocols (e.g., posteroanterior images, obviating lateral radiographs during follow-up). However, tumor biology is highly complex, with the ultimate course being the result of many interacting factors. Furthermore, much is still unclear about scoliosis. Its genetic profile, lifestyle adaptions, and other factors may all influence the development of cancer [21]. The results from this study could not prove that taking radiographs is going to make a difference in females.

There are several limitations to consider in this study. The results of this study were not based on an experimental study design but on observational data after treatment during childhood. Therefore, it is impossible to investigate the causal effects of radiographs and cancer. This uncontrolled study design represents level 3 evidence, and some bias likely exists.

Despite all efforts, there was a selection bias since not all patients could be traced. It is also possible that the questionnaire responders represent a relatively healthy subgroup of the entire scoliosis cohort given the low number of smokers (8,6%) in our cohort compared to the average Dutch population aged 40 to 50 years old with 26% [22]. Radiation is a known risk factor for basal cell carcinoma (BCC) [23,24]. However, BCC is not registered by the IKNL so we could not include it in our analysis. Furthermore, three different sources were reviewed for information regarding radiographic examinations during childhood. However, the risk of missing radiographic examinations remains since this study dealt with charts from more than 20 years ago.

Finally, despite being one of the recent larger studies on this subject, this study was also underpowered. We anticipate that only a limited number of centers worldwide have retained their historical data on radiation use in children, spanning a sufficient timeframe for the comprehensive assessment of its carcinogenic effects. This aspect underscores the valuable and significant nature of these data. These findings hold importance in the broader context of scientific research and can serve as crucial puzzle pieces for future meta-analysis, enabling more robust and adequately powered conclusions. Moreover, the accrued data and unique insights obtained from this cohort, particularly through our meticulous methodology using the SPR, manual data extraction from archival records, and comparisons made during a period when X-ray machines and protocols provided a comparable level of radiation, contribute significantly to the ongoing discussion.

Although no significant increased cancer risk was found, we do support the ALARA principle (‘as low as reasonably achievable’) to limit the radiation dose in children. The ICRP recommends limiting radiation exposure to the levels required to obtain the desired images [25]. The International Atomic Energy Agency (IAEA) has established a guidance level, which is the upper limit of the absorbed dose at each X-ray examination [26]. To date, there is no consensus on the reference level for children for full spine X-rays. The optimal radiation dose required for adequate image quality should be determined to reduce radiation exposure. Furthermore, full spine X-ray examinations using 0.2-mm Cu filters could reduce radiation exposure more than 60% while preserving the image quality [27]. New microdose X-ray machinery with or without reduced radiation protocols could reduce the amount of radiographic radiation doses [26,28,29]. In addition, there is a lack of published literature indicating how often X-rays are necessary during scoliosis surveillance. Despite the systematic underestimation of the Cobb angle, ultrasound imaging of the spinal curve may be an alternative to replace some of the X-rays during the monitoring of curve progression. While this decrease in total radiation dose is expected to reduce the risk of carcinogenesis, the study did not find any evidence to suggest that females undergoing repeated radiographic monitoring for IS were at a higher risk of developing cancer compared to the general population.

## 5. Conclusions

The results of this study do not suggest that female patients treated for idiopathic scoliosis during childhood have an increased risk of cancer later in life. However, clinicians should continue to limit the radiation exposure of children and the frequency and number of radiographic studies for individual patients. While our study wrestled with underpowered constraints, the detailed data collected, the long observation period, and data gathered from a period with radiographic equipment and protocols with comparable radiation rates to the present contribute not only to the ongoing refinement of clinical practice but also lay the groundwork for subsequent more robust meta-analyses, facilitating the generation of well-powered assertions in the future.

## Figures and Tables

**Table 1:** Baseline characteristics of the cohort *n* = 337.

		Total		Observation		Brace		Surgery	
		(*n* = 337)		(*n* = 87)		(*n* = 175)		(*n* = 75)	
Sex									
	Female	298 (88%)		70 (80%)		160 (91%)		68 (91%)	
	Male	39 (12%)		17 (20%)		15 (8.6%)		7 (9.3%)	
Age at follow-up, years									
	Mean, SD	44 ± 6.6		44 ± 5.7		44 ± 6.2		43 ± 8.0	
Year of birth									
	<1960	8 (2.4%)		1 (1.1%)		2 (1.1%)		5 (6.7%)	
	1960–1969	93 (28%)		19 (22%)		53 (30%)		21 (28%)	
	1970–1979	207 (61%)		61 (70%)		110 (63%)		36 (48%)	
	≥1980	29 (8.6%)		6 (6.9%)		10 (5.7%)		13 (17%)	
Age at first radiograph									
	Median, IQR	13 (11–15)		14 (12–16)		13 (11–14)		13 (11–15)	
	<10	48 (14%)		10 (11%)		26 (15%)		12 (16%)	
	10–13	161 (48%)		33 (38%)		98 (56%)		30 (40%)	
	14–18	128 (38%)		44 (51%)		51 (29%)		33 (44%)	
Diagnosis									
	Juvenile	81 (24%)		12 (14%)		44 (25%)		25 (33%)	
	Adolescent	256 (76%)		75 (86%)		131 (75%)		50 (67%)	
Cobbs angle, degrees †									
	<20	41 (12%)		33 (38%)		7 (4.0%)		1 (1.3%)	
	20–29	70 (21%)		24 (28%)		44 (25%)		2 (2.7%)	
	30–39	101 (30%)		18 (21%)		73 (42%)		10 (13%)	
	40–49	65 (19%)		5 (5.7%)		36 (21%)		24 (32%)	
	≥50	60 (18%)		7 (8.0%)		15 (8.6%)		38 (51%)	
Total no. of X-rays									
	Median, IQR	14 (8–19)		6 (4–9)		15 (12–20)		17 (12–24)	
	1–9	111 (33%)		68 (78%)		27 (15%)		16 (21%)	
	10–19	147 (44%)		19 (22%)		99 (57%)		29 (39%)	
	20–29	57 (17%)		0 (0%)		39 (22%)		18 (24%)	
	≥30	22 (6.5%)		0 (0%)		10 (5.7%)		12 (16%)	
No. of X-rays <18 years old									
	Median, IQR	12 (6–17)		5 (3–8)		14 (11–18)		15 (9–22)	
	1–9	129 (38%)		73 (84%)		34 (19%)		22 (29%)	
	10–19	147 (44%)		14 (16%)		106 (61%)		27 (36%)	
	20–29	46 (14%)		0 (0%)		28 (16%)		18 (24%)	
	≥30	15 (4.4%)		0 (0%)		7 (4.0%)		8 (11%)	
Time between X-rays in years									
	Median, IQR	5.8 (3.1–10)		2.6 (1.2–6.8)		7.1 (4.4–10)		7 (3.8–13)	
Age of menarche									
	<12	26 (7.7%)		7 (8.1%)		14 (8.0%)		5 (6.7%)	
	12–14	204 (60%)		48 (55%)		116 (66%)		40 (53%)	
	≥15	21 (6.2%)		2 (2.3%)		12 (6.9%)		7 (9.3%)	
	Unknown	86 (26%)		30 (34%)		33 (19%)		23 (31%)	
BMI (mean, SD)									
	At first radiograph	18 ± 2.7		19 ± 2.5		18 ± 2.6		19 ± 3.1	
	At last radiograph	20 ± 2.9		20 ± 2.6		20 ± 2.8		21 ± 3.2	
Smoking *									
	Never	246 (74%)		62 (71%)		126 (73%)		58 (78%)	
	Past	41 (12%)		14 (16%)		19 (11%)		8 (11%)	
	Occasional	19 (5.7%)		4 (4.6%)		11 (6.4%)		4 (5.4%)	
	Current	27 (8.1%)		7 (8.0%)		16 (9.3%)		4 (5.4%)	
Deceased									
	<40	4 (1.2%)		0 (0%)		3 (1.7%)		1 (1.3%)	
	40–49	7 (2.1%)		1 (1.1%)		3 (1.7%)		3 (4.0%)	
	≥50	3 (0.9%)		0 (0%)		0 (0%)		3 (4.0%)	

† maximal recorded degrees. * Not available for deceased patients. Abbreviations: IQR interquantile range; SD standard deviation.

**Table 2:** Types of cancer.

	Total No. of Patients	Female Sex	Age at Diagnosis in Years *
Breast cancer †	4 (1.2%)	4	49 (45–53)
Melanoma	3 (0.9%)	3	39 (29–44)
Ovarian cancer	2 (0.6%)	2	35
Cervical cancer	3 (0.3%)	3	49 (47–50)
Hodgkin’s lymphoma	1 (0.3%)	1	37
Lung cancer	1 (0.3%)	1	26
Brain cancer (Astrocytoma)	1 (0.3%)	1	26
Renal cell carcinoma	1 (0.3%)	1	33 (46)
Anal cancer	1 (0.3%)	0	47
Total	17 (5.6%)	16	39 (31–46)

* Multiple values (>2) were presented with median and interquantile ranges. † Two patients who are not listed in the table had breast cancer recurrence.

**Table 3:** Observed and expected cancer rates for women.

Age	National Population Cancer Rates (%)	Number of Women in Our Cohort	Observed Cancer *	Expected Cancer Rates (Rounded)
0	*-*	*-*	*-*	*-*
1–5	*-*	*-*	*-*	*-*
6–10	*-*	*-*	*-*	*-*
11–15	0.06	0	0	0.00
16–20	0.17	2	0	0.00
21–25	0.35	1	0	0.00
26–30	0.69	7	3	0.05
31–35	1.31	11	2	0.14
36–40	2.29	64	5	1.47
41–45	3.77	83	1	3.13
46–50	6.06	96	4	5.82
51–55	9.29	24	0	2.23
56–60	13.38	8	0	1.07
61–65	18.72	2	0	0.37
Total		298	15 (5.0%)	14.3 (4.8%)

Prevalence rates from the IKNL were taken from age 10 because 75% of our cohort had adolescent scoliosis (i.e., scoliosis diagnosis received 10–18 years of age) with a mean age at diagnosis of 11.4 years (±2.9). * Only the initial event is included if a person developed cancer multiple times during our follow-up.

## Data Availability

The data presented in this study are available on request from the corresponding author.
